# Ballistic Performance of Raffia Fabric-Reinforced Epoxy Composites as an Intermediate Layer in Multilayered Armor Systems

**DOI:** 10.3390/polym17212827

**Published:** 2025-10-23

**Authors:** Douglas Santos Silva, Raí Felipe Pereira Junio, Leticia dos Santos Aguilera, Sergio Neves Monteiro, Marcelo Henrique Prado da Silva

**Affiliations:** 1 Department of Materials Science, Military Institute of Engineering—IME, Praça General Tibúrcio, 80, Praia Vermelha, Urca, Rio de Janeiro 22290-270, RJ, Brazil; raivsjfelipe@ime.eb.br (R.F.P.J.); sergio.neves@ime.eb.br (S.N.M.); marceloprado@ime.eb.br (M.H.P.d.S.); 2 Materials Department, Polytechnic Institute, State University of Rio de Janeiro—UERJ, Rua Bonfim, 25, Vila Amélia, Nova Friburgo 28625-570, RJ, Brazil; leticia.aguilera@iprj.uerj.br

**Keywords:** *Raphia vinifera*, raffia fabric, epoxy composite, ballistic test, multilayered armor

## Abstract

This study investigates the ballistic performance of epoxy matrix composites reinforced with raffia fabric, aiming to evaluate their potential as the second layer in multilayered armor systems (MAS), replacing conventional synthetic aramid (Kevlar™) laminates. Composite plates with different volumetric fractions of raffia fabric (10, 20, and 30%) were manufactured and integrated with a ceramic front layer (Al_2_O_3_/Nb_2_O_5_) in MAS structures, which were then subjected to ballistic impact tests using high-energy 7.62 mm caliber ammunition. The backface signature (indentation depth) measured in ballistic clay, used as a human body simulant, showed that only the 10% raffia-reinforced composite (ER10) met the National Institute of Justice (NIJ 0101.06) safety threshold of 44 mm. Higher raffia contents (20% and 30%) led to increased indentation, compromising ballistic integrity. Scanning electron microscopy (SEM) of the fractured surfaces revealed typical energy dissipation mechanisms, such as fiber rupture, fiber pull-out, and interfacial delamination. The results indicate that raffia fabric composites with 10% fiber content can serve as a cost-effective and sustainable alternative to Kevlar™ in personal armor applications, while maintaining compliance with ballistic protection standards.

## 1. Introduction

Protective materials and ballistic armor play a critical role in safeguarding human lives against threats ranging from electromagnetic interference to high- and low-velocity projectiles originating from rifles, machine guns, handguns, and other advanced weaponry frequently encountered in domestic conflicts and national defense scenarios [1]. To ensure effective protection, ballistic armors must combine mechanical efficiency with lightweight design, a requirement that has driven the adoption of fiber-reinforced composites. These materials stand out due to their high specific strength and stiffness, excellent strain-to-failure ratios, and tailored mechanical behavior, which make them ideal candidates for structural and ballistic applications [2].

Traditionally, the development of ballistic composites has relied on synthetic high-performance fibers such as aramid (e.g., Kevlar™, Twaron, Arnhem, The Netherlands), ultra-high molecular weight polyethylene (e.g., Dyneema^®^, Geleen, The Netherlands, Spectra^®^, Gurugram, India), carbon, and glass fibers. While these materials offer excellent protective capabilities, they are often derived from non-renewable sources, entail high production costs, and pose long-term environmental concerns due to their poor biodegradability and high energy demand during manufacturing [3].

In this context, natural lignocellulosic fibers (NLFs) have emerged as a sustainable and eco-friendly alternative to synthetic reinforcements. Sourced from abundant renewable resources, natural fibers such as jute, sisal, hemp, flax, and more recently raffia, are biodegradable, lightweight, non-toxic, and exhibit low abrasiveness to processing equipment [4,5,6,7,8,9,10]. Moreover, their incorporation into polymeric matrices contributes to reducing the carbon footprint and overall environmental impact of advanced materials while offering cost-effective solutions for large-scale production [11,12,13,14,15,16,17]. From a socioeconomic standpoint, the valorization of agricultural by-products and the use of plant-derived fibers support rural economies and promote the circular bioeconomy, especially in developing countries rich in biodiversity and natural resources.

Ballistic composites reinforced with natural fibers are now gaining momentum as viable alternatives to traditional synthetic multilayer armor systems. Recent investigations have highlighted their promising mechanical performance, energy absorption capacity, and impact resistance, prompting their consideration for use in body armor, helmets, protective shields, and vehicle panels [18,19,20,21,22,23,24]. Research efforts are increasingly focused not only on mechanical and thermal characterization, but also on failure mechanisms, fiber-matrix interaction, moisture sensitivity, and long-term durability in varying environmental conditions [25,26,27,28,29,30].

Among the various natural fibers under exploration, raffia, extracted from the leaves of *Raphia vinifera*, a palm species native to tropical regions, has shown potential for technical applications beyond its traditional use in ropes, rugs, and handicrafts. Recent thermochemical and morphological studies have identified raffia fiber fabric as a candidate reinforcement for polymer-based composites, thanks to its balanced composition of cellulose, hemicellulose, and lignin, combined with favorable thermal stability and surface characteristics [31].

Raffia fabric (*Raphia vinifera*) was chosen as the reinforcement based on (i) local availability in the Amazon region in a woven form compatible with laminate processing; (ii) low density and a fibrillated, rough surface that favors mechanical interlocking and energy-dissipation mechanisms (fiber pull-out and progressive debonding); (iii) balanced lignocellulosic composition and adequate thermochemical stability for short-duration ballistic events; (iv) promising preliminary ballistic results reported by our group for raffia/epoxy laminates in both stand-alone and MAS configurations; and (v) sustainability/cost advantages relative to imported aramid or UHMWPE fabrics. While other natural fibers (e.g., jute, hemp, flax, sisal) have been widely studied, raffia remains comparatively underexplored in MAS, and the present work deliberately focuses on establishing how raffia fiber volume fraction (10–30 vol%) influences ballistic trauma under a consistent ceramic/front-layer configuration [19,25].

Building upon this foundation, the present study offers an unprecedented evaluation of the ballistic performance of epoxy matrix composites reinforced with woven raffia fabric, specifically applied as a second layer in multilayer armor systems (MAS). The primary goal is to assess their feasibility as a sustainable alternative to synthetic aramid laminates (Kevlar™), which dominate the personal protection market but are costly, non-biodegradable, and dependent on petrochemical routes. The novelty of this work lies not only in the material configuration employed, woven raffia fabric in MAS structures, but also in the rigorous ballistic testing protocol using high-energy 7.62 mm ammunition, following the standards established by the National Institute of Justice. Particular emphasis is given to evaluating the backface signature (BFS) or trauma depth in ballistic clay, with a critical limit of 44 mm used as the benchmark for acceptable protection.

Furthermore, the study integrates comprehensive microstructural and failure analyses via Scanning Electron Microscopy (SEM) to elucidate the energy dissipation mechanisms involved, such as fiber rupture, pullout, and interfacial delamination, thus correlating macro-level ballistic performance with meso- and micro-scale structural features. These investigations provide valuable insights for optimizing composite design and hybridization strategies in future developments.

By bridging technical performance with environmental responsibility and socioeconomic viability, this research contributes to the growing field of sustainable protective systems, laying the groundwork for innovative applications in defense, law enforcement, and civilian safety with reduced ecological impact and lower material costs.

## 2. Materials and Methods

### 2.1. Materials

#### 2.1.1. Raffia Fabric (*Raphia vinifera*)

**Figure 1:**
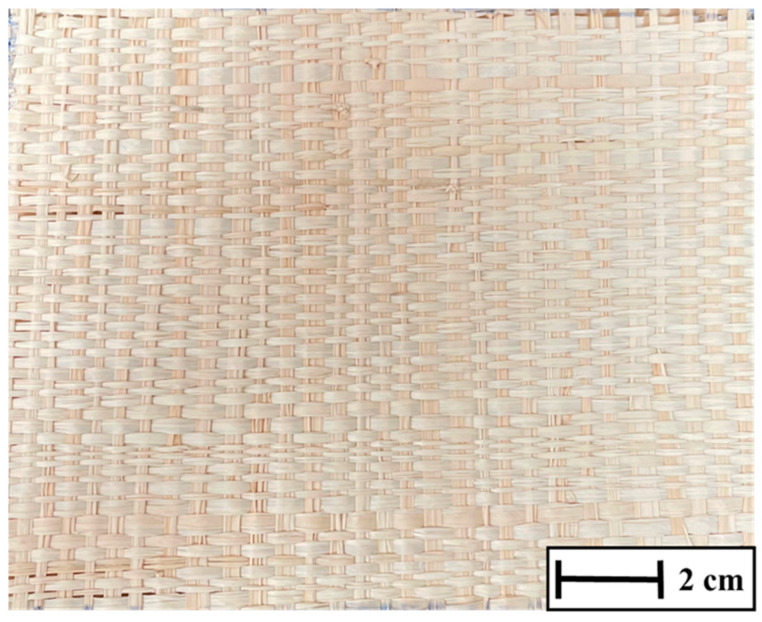
Raffia fiber fabric.

The raffia fabric used in this work was purchased from a store in the city of Belém, Pará, Brazil. The raffia fabric, as purchased, is illustrated in Figure 1. The choice of raffia fabric was guided by its consistent availability in fabric form, low density (0.95 g/cm^3^) [31], surface fibrillation/roughness that enhances mechanical anchoring with epoxy, and prior results indicating NIJ-compliant BFS at lower volume fractions in raffia/epoxy laminates under comparable impact conditions. These factors, coupled with lower cost and renewable sourcing, justified prioritizing raffia over other natural fibers or synthetic fabrics for the present parametric study of MAS intermediate layers.

#### 2.1.2. Epoxy Resin

The matrix material for the composite plate consisted of commercial ether-type epoxy resin, diglycidyl bisphenol A (DGEBA), cured with triethylene tetramine (TETA) at a stoichiometric ratio of 13 parts hardener to 100 parts resin. It was produced by Dow Chemical in Brazil and provided and distributed by EPOXY FIBER Ltd. (Rio de Janeiro, RJ, Brazil).

#### 2.1.3. Aramid Fabric

The material utilized in this project was provided by LFJ Armoring Trade and Services S.A. (Conquext), featuring an S745 weave and a weight of 460 g/m^2^, presented as 8-layer panels bonded with chloroprene rubber (NEOKV08).

#### 2.1.4. Ceramic Samples

The initial phase of processing the ceramic samples involved preparing the powder blend. Alumina powder (700 g, 94.5% wt.), niobium powder (29.15 g, 3.94% wt.), and liquid PEG binder (11.3 g, 1.53% wt.) were blended in a ball mill using alumina balls, model MA 500, for 8 h. Following the grinding process, the blend was placed in an oven at 60 °C and dried for 48 h. Subsequently, the powder was deagglomerated with a mortar and pestle, and it was subsequently passed through a sieve featuring an opening of 0.355 mm.

Following the sieving process, the ceramic powder (100 g) was compacted in a hexagonal mold, created by two punches and a movable jacket. A load of 12 tons, which is equal to 30 MPa, was utilized, as outlined in the literature, using an SKAY hydraulic press. The “green” ceramic tablets were fired in an INTI furnace, model FE 1700, at the IME Ceramic Materials Laboratory. The sintering method applied is well known for producing alumina samples with excellent densification and is outlined below:

- Heating from 25 °C to 158 °C at a speed of 1 °C/min;
- Maintaining a temperature of 158 °C for 1 h;
- Heating from 158 °C to 375 °C at a speed of 1 °C/min;
- Raising the temperature from 375 °C to 1000 °C at a speed of 8 °C/min;
- Heating from 1000 °C to 1400 °C at a speed of 5 °C/min;
- Sintering maintained at 1400 °C for 3 h, followed by cooling in the furnace.

The ceramic processing parameters were fixed by combining literature guidance and in-house optimization [32] with equipment constraints. The powder composition targeted ~96 wt.% Al_2_O_3_ and ~4 wt.% Nb_2_O_5_ after binder removal because 3–5 wt.% Nb_2_O_5_ in our prior trials promoted densification without abnormal grain growth or excessive liquid formation. The PEG binder level (~1.5 wt.%) was the lowest content that ensured granule cohesion and mold release while avoiding large organics loads during debinding. Ball milling for 8 h (alumina media) was the minimum time that broke soft agglomerates and homogenized Nb_2_O_5_ distribution, as verified by flow and green density repeatability. Drying at 60 °C/48 h prevented premature binder degradation, and sieving at 0.355 mm produced a granule size window that reduced lamination and edge chipping in the hexagonal die. The compaction pressure (~30 MPa) was set by press capacity and by the need to reach green densities typically in the 55–60% T.D. range without inducing lamination cracks. The sintering schedule adopted slow debinding ramps (1 °C·min^−1^ to 375 °C) to avoid internal pressure build-up, followed by intermediate heating (8 °C·min^−1^ to 1000 °C) and a densification ramp (5 °C·min^−1^ to 1400 °C) with a 3 h dwell to achieve stable neck growth and pore shrinkage. Furnace tolerances were ±2 °C in dwell and ±0.2 °C·min^−1^ in ramps. With these settings, tiles consistently reached an average sintered density of 3.53 g·cm^−3^ and ~88% relative density, matching the performance envelope previously reported by our group [32] and providing mechanically sound front layers for MAS assembly.

The initial three phases of this pathway led to the removal of the organic binder; consequently, the material’s composition changed to 96% alumina and 4% niobium. The material exhibited an average densification of 88.1% during sintering, with a mean sintered density of 3.53 g/cm^3^ [32]. Following the sintering process, the samples were utilized to create the MAS meant for ballistic evaluation.

### 2.2. Methods

#### 2.2.1. NIJ Standard 0101.06

NIJ 0101.06 is a standard that outlines the essential performance criteria and testing methods for the ballistic protection of body armor utilized by law enforcement and corrections personnel [33]. The standard aims to guarantee that body armor offers reliable defense against various ballistic threats while also reducing the likelihood of injury to the user. To accomplish this, the standard encompasses requirements for evaluations like residual velocity and backface signature (BFS). The residual velocity test assesses the speed of a bullet once it has gone through the armor, whereas the BFS test evaluates the depth of the mark left on the clay backing material when a bullet impacts the armor’s surface. The highest permissible residual velocity and indentation are defined for every protection level.

#### 2.2.2. Composite Fabrication

The epoxy–raffia composites were fabricated with reinforcement contents of 10, 20, and 30 vol%. These specific fiber fractions were selected based on two main criteria: (i) processability and impregnation limits reported in the literature for lignocellulosic fiber–epoxy systems [18,19,20,21,22,23,24], and (ii) the need to establish a comparative range between a lower reinforcement level (10%), an intermediate level (20%), and a higher level (30%) in order to evaluate the influence of fiber content on ballistic performance. Preliminary studies have indicated that fractions above 30% generally result in poor resin infiltration, void formation, and weak interfacial bonding, which compromise structural integrity [25,31].

Prior to processing, the raffia fabric was oven-dried at 60 °C for 24 h to reduce moisture content and enhance fiber–matrix adhesion; no chemical surface treatments were used in this work. The composite plates were then produced by compression molding in a metallic mold with an internal volume of 214.2 cm^3^ (15 × 12 × 1.19 cm^3^). The raffia fabrics were manually arranged in the mold and impregnated with the epoxy mixture (DGEBA/TETA, 100:13 phr). A uniaxial hydraulic press was applied under a constant load of 5 tons (≈12 MPa) for 8 h, which ensured uniform consolidation of the laminates, reduced the amount of porosity, and improved matrix impregnation of the raffia fabrics.

**Figure 2:**
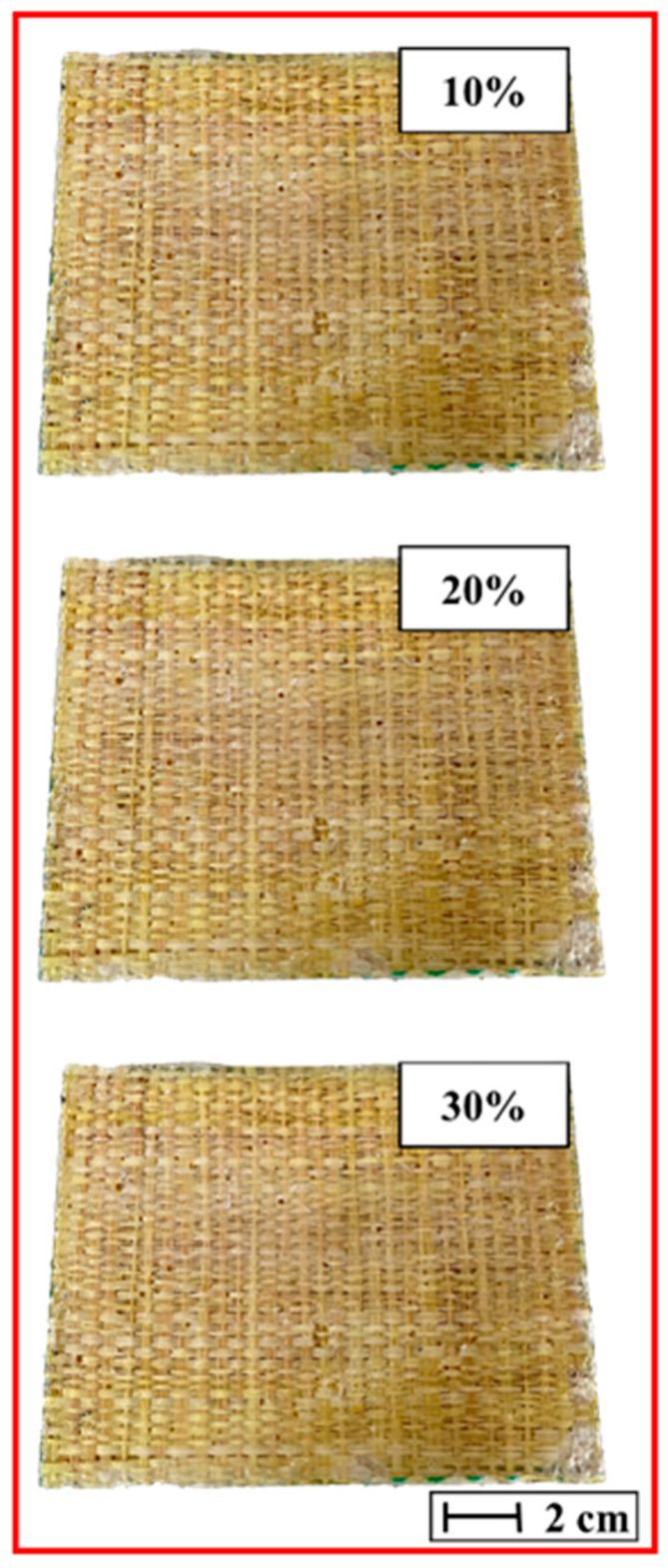
Epoxy/raffia laminates with 10% (ER10), 20% (ER20), and 30% (ER30) fiber volume fractions. Images are provided for identification only; macroscopic appearance is not expected to reveal Vf differences. A 2 cm scale bar is shown for reference. Quantitative descriptors (mass/area and thickness) used to document Vf are reported in the Methods and Results.

The resulting plates were designated according to their raffia volume fraction (ER10, ER20, and ER30) and are shown in Figure 2. The nomenclature used is shown in Table 1. The chosen processing route provided consistent, defect-minimized laminates suitable for ballistic evaluation, while also highlighting the influence of reinforcement level on the trade-off between energy absorption and structural cohesion.

The three fiber volume fractions were chosen to span the processability envelope of raffia/epoxy laminates manufactured by hand lay-up and consolidated under constant load, while preserving statistical power in ballistic testing (*n* = 5 shots per level). Based on pilot plates and on reported wet-out limits for lignocellulosic fibers in epoxy, 10 vol% represents a lower bound that still activates fiber-mediated energy-dissipation mechanisms, 20 vol% is an intermediate, well-wetted condition, and 30 vol% is an upper bound beyond which voiding and resin-starved regions tend to increase. Using three well-separated levels allows a one-factor ANOVA with adequate replication under cost/safety constraints inherent to NIJ-type ballistics tests. Finer 5% increments would either require a substantially larger number of MAS targets or reduce replicates per level, compromising inference; therefore, the present work was designed as a coarse-grid exploration to identify the viable region, to be narrowed in future studies.

Because the three laminates share the same nominal plate geometry and weave, visual inspection is insufficient to distinguish fiber volume fraction (Vf). In practice, the target Vf (10/20/30 vol%) was achieved by pre-weighing the dry fabric stack and metering the resin to fill the mold’s internal volume, followed by pressing under fixed load/time. After curing, we recorded panel mass (±0.01 g) and thickness (±0.01 mm) and reported the areal density (mass/area) together with thickness. These quantitative descriptors, rather than macroscopic appearance, document the intended Vf levels used for ballistic testing.

#### 2.2.3. Backface Signature (BFS) Tests

The ballistic evaluation focused on measuring the backface signature (BFS), which corresponds to the indentation depth in a ballistic clay witness block placed behind the multilayered armor system (MAS). The clay simulates the mechanical response of human soft tissue and provides a quantitative parameter for trauma assessment.

The tests were conducted in accordance with NIJ Standard 0101.06 [33], which establishes the most recent and internationally recognized requirements for body armor certification. This standard was selected instead of the previous NIJ 0101.04 because it provides stricter guidelines, harmonized testing procedures, and greater reliability in evaluating non-perforating trauma. In particular, NIJ 0101.06 defines a maximum BFS of 44 mm, above which the armor is considered to provide insufficient protection, even in the absence of projectile perforation.

Before testing, the Roma Plastilina Nº 1 clay blocks were conditioned at 29 ± 2 °C for at least 3 h to achieve the plasticity and density required by the standard and to eliminate entrapped air bubbles. The MAS samples were assembled by bonding the ceramic tile (Al_2_O_3_/Nb_2_O_5_), the raffia/epoxy composite plate, and the aramid fabric backing using a fast-curing polyurethane adhesive. The assembled targets were positioned in front of the clay witness block, and each was impacted by a 7.62 mm projectile fired at a velocity consistent with NIJ Level III requirements.

Five shots were fired for each condition. After each shot, the BFS was measured at the point of maximum indentation using a laser depth sensor with ±0.1 mm precision. A BFS below 44 mm was considered satisfactory, while values above this threshold indicated unacceptable trauma levels according to NIJ 0101.06.

**Figure 3:**
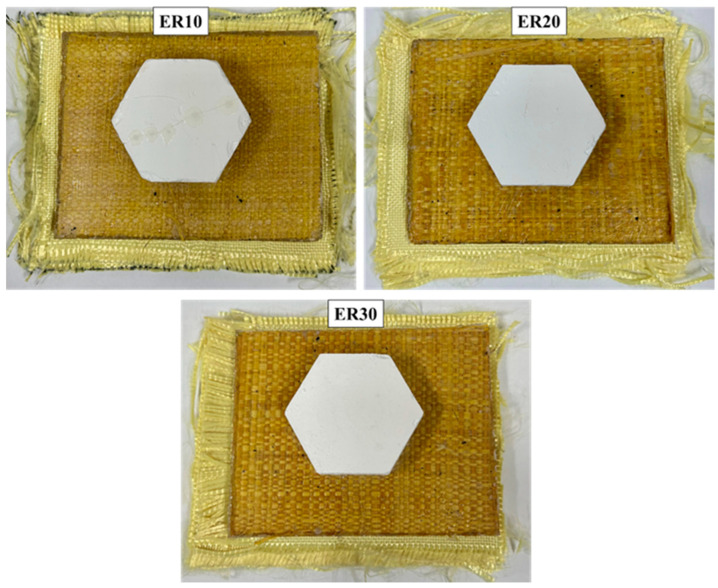
Multilayer Armor Systems (MAS) reinforced with raffia fabric produced with different fiber volume fractions: ER10 (10%), ER20 (20%), and ER30 (30%).

The test samples for the multilayer ballistic evaluation were created by bonding the individual components (ceramic tile, composite, aramid fabric) using fast-drying polyurethane (PU) adhesive, as illustrated in Figure 3.

**Figure 4:**
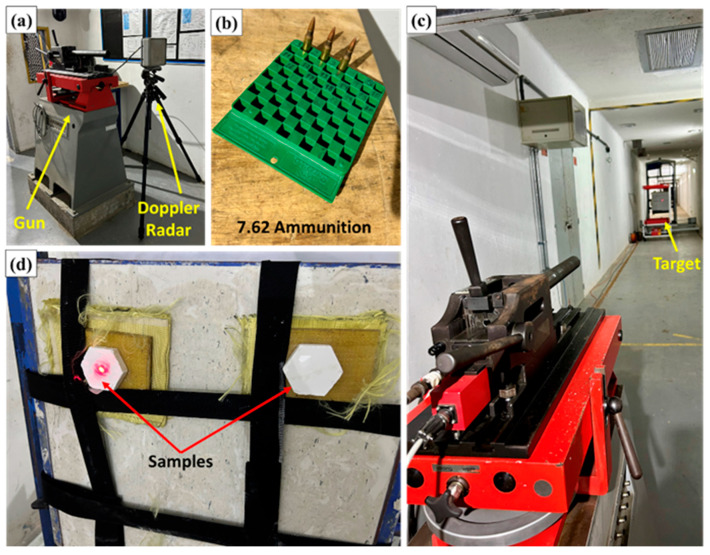
Experimental setup used in ballistics tests with 7.62 mm ammunition: (**a**) test cannon coupled to Doppler radar to measure projectile velocity; (**b**) 7.62 mm ammunition cartridges; (**c**) view of the shooting range showing the alignment between weapon and target; (**d**) impact panel with composite samples fixed in front of the ballistic clay to evaluate the post-impact response.

Figure 4 shows the experimental setup used in the ballistics tests with 7.62 mm ammunition.

#### 2.2.4. Doppler Radar Measurement and Definition of Radial Velocity

**Figure 5:**
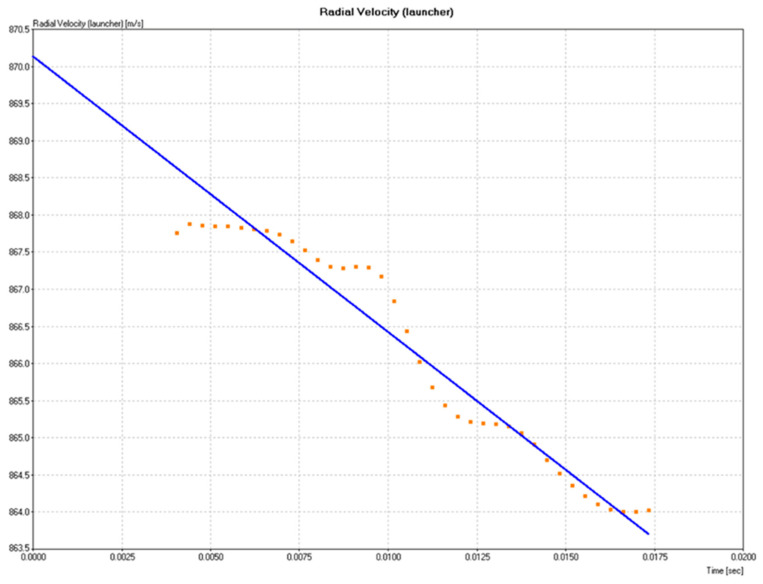
Time history of the projectile radial velocity $v_{r}$ (line-of-sight component measured by Doppler radar, $v_{r}\approx v$ in our coaxial setup) during the ballistic test of MAS-ER10. The initial near-constant segment reflects ceramic break-up, followed by a marked decrease associated with energy absorption in the raffia/epoxy and textile backing. The negative slope $dv_{r}/dt$ represents deceleration, implying a resisting force $F_{res}=m{dv}_{r}/dt$; the reduction in $v_{r}$ quantifies energy dissipation by the armor, $∆E=\frac{1}{2}m({v_{i}}^{2}-{v_{r}}^{2})$, which correlates with lower BFS.

In this study, the projectile speed was tracked with a continuous-wave Doppler radar aligned with the firing line. The radar reports the radial velocity $v_{r}$, defined as the line-of-sight (LOS) component of the projectile velocity vector with respect to the radar. Formally, if R(t) is the instantaneous radar–projectile range and θ is the angle between the projectile velocity vector v and the radar LOS, then according to Equation (1).

(1) $$v_{r}=\frac{dR}{dt}=v\;Cos\theta$$

where v= ‖v‖. By convention in this work, positive $v_{r}$ denotes motion toward the radar; units are in m·s^−1^. Because the test cannon and radar were coaxially aligned with the target, θ was kept small during shots, so $v_{r}\approx v$ throughout the measured interval. The initial plateau in the $v_{r}(t)$ record (Figure 5) corresponds to the projectile transit and ceramic break-up, whereas the subsequent monotonic decrease reflects energy dissipation in the composite back-up layer (raffia/epoxy) and the textile backing. This LOS definition explains why the plotted “radial velocity” directly represents the effective projectile speed in our setup and supports the interpretation of the deceleration phases discussed in Section 3.1. Thus, the armor’s resisting action is read directly from the deceleration of $v_{r}(t)$ and the associated reduction of $v_{r}$, which together indicate the force and energy absorbed by the target during impact.

#### 2.2.5. Statistical Analysis

Statistical tests are fundamental tools for guiding data-driven decisions. Among them, ANOVA and Tukey’s test are commonly employed when comparing multiple groups. In this study, ANOVA was used as the statistical method to evaluate the ballistic performance of the materials tested. The significance level (α) represents the probability of committing a type I error, meaning the null hypothesis (H_0_) is rejected even though it is true. For ANOVA and Tukey’s test, a typical significance threshold is 5% (α = 0.05), which implies a 5% risk of identifying a difference between groups that does not actually exist. The reliability of the test, also referred to as the confidence level, is defined as 1 − α. Thus, with a 5% significance level, the confidence level is 95%. In practice, this indicates that if the experiment were repeated numerous times, similar results would be expected in 95% of the trials. In this work, a 5% significance level (95% confidence) was adopted, enabling the assessment of how different fiber contents influenced the composite properties. The Tukey test was applied to perform pairwise comparisons of the mean values obtained for each treatment condition (fiber percentage). From these results, it was possible to determine whether to reject or accept the hypothesis of equality among the means, based on the minimum significant difference (*d.m.s.*), as expressed in Equation (2).

(2) $$d.m.s.=q.\sqrt{\frac{QMR}{r}}$$

where

*q* represents the studentized range (a tabulated value that depends on the degrees of freedom, the residual, and the number of treatments);
*QMR* corresponds to the mean square of the residual;
*r* denotes the number of replications for each treatment.

Through this approach, it was possible to assess, both quantitatively and qualitatively, the comparative influence of the raffia fiber volume fraction used in the composites, allowing the identification of the treatment that yielded the most favorable results. Descriptive statistics (mean, sample SD with n − 1) were computed per NIJ shot condition (*n* = 5).

#### 2.2.6. Fracture Surface Analysis

The analysis of the main failure mechanisms associated with fracture was carried out after the ballistics tests of the composite panels, considering both macro- and microscopic scales. The fractured specimens were examined using a scanning electron microscope (SEM), model Quanta FEG 250 FEI (Thermo Fisher Scientific, Waltham, MA, USA), operating with secondary electrons at 10 kV.

## 3. Results and Discussions

### 3.1. Back-Face Signature (BFS) Depth Testing of the Multilayered Armor System (MAS)

The BFS evaluations sought to determine the viability of employing epoxy–raffia composites as an intermediary layer in an MAS. The trauma results acquired following ballistic impact for the assessed MAS are shown in Table 2. As the three laminates have the same geometry and weave, no visible differences are expected in the macro photographs (Figure 2); therefore, areal density and thickness are reported and used to distinguish the ER10/ER20/ER30 conditions, alongside their BFS outcomes.

Table 2 presents the results obtained in the ballistic test with measurement of residual trauma, quantified by the indentation depth in ballistic clay, for epoxy matrix composites reinforced with raffia fabric in the proportions of 10% (ER10), 20% (ER20), and 30% (ER30). This test followed the requirements established by the NIJ 0101.06 standard, which establishes that the maximum acceptable depth of deformation in the ballistic simulant (ballistic clay) is 44 mm, above which the armor is considered ineffective, regardless of the occurrence of perforation.

It can be observed that the ER10 composite (10% raffia) presented satisfactory performance, with an average indentation depth of 38.34 mm and a standard deviation of 0.29 mm, remaining below the established limit. This result demonstrates high efficiency in dissipating the kinetic energy of the projectile, ensuring adequate protection against residual trauma. The low dispersion of the results also suggests good uniformity in the distribution of the fibers and adequate matrix-fiber interfacial adhesion in this condition. This superior trauma control at 10 vol% is consistent with the energy-dissipative failure mechanisms observed in SEM (fiber pull-out with fibrillation, mixed-mode matrix fracture, and resin tags), whereas higher fractions trend toward delamination-dominated and plugging responses (see Section 3.3).

In contrast, the ER20 composite (20% raffia) had an average depth of 46.82 mm, with a standard deviation of 1.73 mm, significantly exceeding the normative limit. This result shows that, for this fiber proportion, the material does not meet the ballistic performance criteria for trauma control. The increase in both the average and variability of the indentation values suggests the formation of internal defects, such as voids and poor impregnation, in addition to fragility at the matrix-fiber interface. These factors compromise the structural integrity of the laminate, favoring delamination and interlaminar failure modes under high-velocity impact.

The ER30 composite (30% raffia) showed the worst performance among the evaluated groups, with an average depth of 58.40 mm and a standard deviation of 0.63 mm. This result demonstrates that, in this condition, the increase in the volumetric fraction of fibers severely compromises the material’s ability to dissipate ballistic energy. The excessive accumulation of fibers, combined with the low wettability and interfacial compatibility with the epoxy matrix, results in the generation of high stress concentrations, which are insufficiently distributed, culminating in localized failures and a significant increase in deformation in the soft tissue simulant.

In general, there is a clear tendency for ballistic performance to decrease in terms of residual trauma control with the increase in the volumetric fraction of raffia fabric. This behavior is consistent with the specialized literature, which indicates that natural composites, when not subjected to interfacial compatibility treatments or when processed with high fiber contents, tend to present greater void formation, deficiencies in impregnation, and increased delamination mechanisms, all of which are detrimental to the efficiency in absorbing ballistic impact energy.

**Figure 8:**
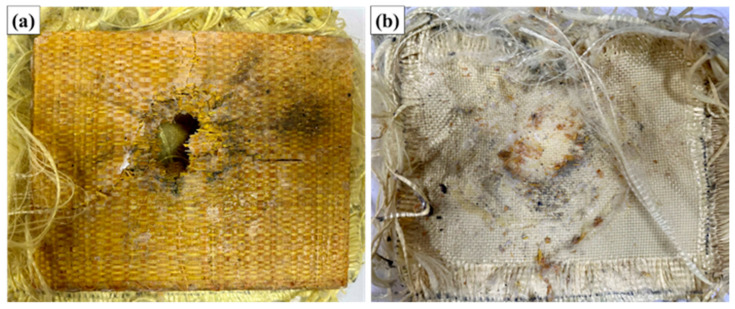
Appearance of the MAS with 20% fiber volume (ER20) after ballistic testing with 7.62 mm ammunition: (**a**) impact face; (**b**) internal face.

**Figure 9:**
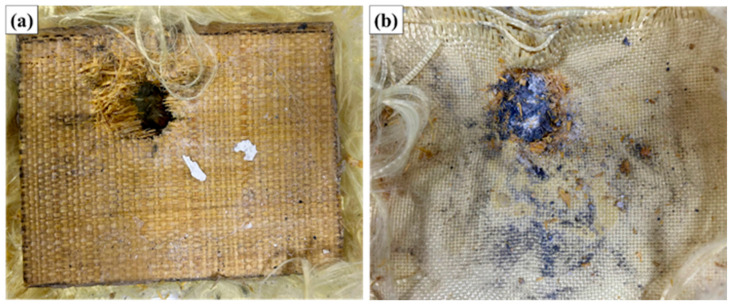
Appearance of the MAS with 30% fiber volume (ER30) after ballistic testing with 7.62 mm ammunition: (**a**) impact face; (**b**) internal face.

**Figure 10:**
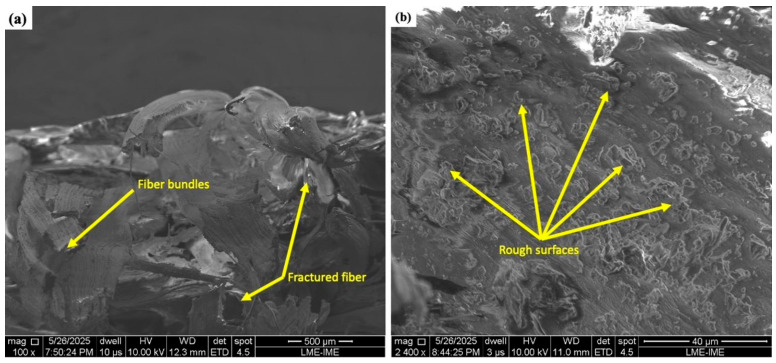
SEM micrographs of the MAS with raffia fabric (ER10): (**a**) 100× magnification; (**b**) 2400× magnification.

The disproportionate increase in BFS from 20% to 30% raffia is consistent with a transition from a “well-wetted, fiber-assisted” regime to a resin-starved, void-susceptible regime. At higher Vf, the reduced free resin limits matrix crack-bridging and stress redistribution, while tighter bundle packing/percolation promotes inter-bundle debonding and early interlaminar shear. Incomplete wet-out at ER30 increases the size/connectivity of voids that act as stress concentrators, facilitating matrix cleavage and delamination. Concomitantly, the stiffness–mass mismatch among the ceramic front, the raffia/epoxy layer, and the textile backing escalates, magnifying through-thickness shear and favoring plugging-type failure rather than controlled fiber pull-out/sliding. This shift in failure mode explains the sharp rise in trauma depth between ER20 and ER30 and is consistent with the macroscopic damage patterns in Figures 8 and 9 and the interfacial features visible in Figure 10.

Therefore, the ER10 composite is the only material approved according to the criteria of the NIJ 0101.06 standard for ballistic trauma control, while ER20 and ER30, although they have resisted perforation, do not meet the safety requirements related to the dissipation of impact energy, being considered unsuitable for ballistic applications without the adoption of modifications in the formulation or in the manufacturing process.

The graph presented in Figure 5 shows the variation in the radial velocity of the projectile as a function of time during the ballistic test carried out with the ER10 composite, which contains 10% by volume of raffia fabric as reinforcement in an epoxy matrix. For clarity, the “radial velocity” reported here is the Doppler radar line-of-sight component of the projectile speed ($v_{r}=v\;Cos\theta$); due to coaxial alignment, $v_{r}$ effectively represents the projectile speed in our experiments (see Section 2.2.4).

The initial velocity recorded was approximately 870 m/s, a value consistent with high-caliber ammunition (7.62 mm) used in armor testing. The blue curve represents the expected linear trend for projectile deceleration in an idealized system, while the orange points correspond to the experimental data obtained during impact.

It can be seen that, in the first instants (up to approximately 0.007 s), the data remain almost constant, which indicates the initial penetration phase, in which the projectile breaks through the frontal ceramic layer of the multilayer system. From approximately 0.010 s onwards, the data show a sharp linear drop, demonstrating that the projectile began to decelerate significantly after interacting with the second layer of the armor, composed of the ER10 composite. This region corresponds to the action of the reinforced polymeric material, whose main function is to absorb energy and contain fragments after the initial impact.

The continuous deceleration observed to approximately 864 m/s, over approximately 0.017 s, demonstrates the ability of the ER10 composite to dissipate kinetic energy efficiently. This characteristic, combined with the trauma depth measured in ballistic clay below the 44 mm limit stipulated by NIJ 0101.06, confirms that the composite with 10% raffia offers satisfactory ballistic performance. Therefore, the data reinforce the viability of using ER10 as a sustainable replacement for Kevlar™ in light armor applications, both from a mechanical and regulatory standpoint.

In our coaxial setup, the Doppler-measured radial velocity $v_{r}$ effectively equals the projectile speed. The slope of the $v_{r}(t)$ curve (negative $dv_{r}/dt$) is the deceleration imposed by the target; via Newton’s second law, the corresponding resisting force is $F_{res}=mdv_{r}/dt$. The energy absorbed by the multilayer system during transit follows the work–energy relation,

$$∆E=\int F_{res}dS=m\int vdv=\frac{1}{2}m({v_{i}}^{2}-{v_{r}}^{2})$$

Consequently, a steeper and longer deceleration segment and a lower exit $v_{r}$ denote greater energy dissipation by the ceramic/intermediate/textile layers and typically correspond to smaller BFS. The ER10 record in Figure 5 illustrates this behavior, consistent with its NIJ-compliant trauma depth.

The epoxy composite reinforced with raffia fabric at a volume fraction of 10% (ER10) showed satisfactory ballistic performance, meeting the indentation depth limit established by NIJ 0101.06 (≤44 mm), which demonstrates its viability as a functional material in the second layer of multilayer armor systems (MAS), traditionally occupied by synthetic fiber laminates such as Kevlar™. Based on this result, it is possible to point out specific practical applications for the developed material, such as its use in personal ballistic vests intended for military or police use, where it can be incorporated between the frontal ceramic layer (Al_2_O_3_/Nb_2_O_5_) and the interface with the user’s body.

Furthermore, the ER10 composite can be applied in modular protection panels for light armored vehicle cabins, in replaceable ballistic plates (trauma plates) attached to flexible vests, and in civil protection devices such as backpacks and safety cases. The performance of ER10 as a projectile residual energy dissipator makes it particularly promising in applications that require lightness, ballistic efficiency, and sustainability. Additionally, its use can be extended to experimental tests and academic developments as a reference for lignocellulosic composites in comparative studies of replacing synthetic fibers with natural ones.

From an industrial point of view, some important limitations can be identified in this work. The first concerns the reproducibility and uniformity of the composite reinforced with raffia fabric, since natural fibers present intrinsic variations in their morphology, chemical composition (cellulose, lignin, and hemicellulose contents), and mechanical properties, which can compromise large-scale quality control. To mitigate this limitation, it is recommended to standardize the fibers through surface treatments (such as controlled alkalization) and prior selection based on morphometric and structural criteria, in addition to the implementation of rigorous protocols for traceability of origin and processing.

Another limitation is the interfacial adhesion between the epoxy matrix and the raffia fabric, especially in composites with higher fiber content (ER20 and ER30), where failures such as delamination and fiber pullout were observed. To overcome this challenge, chemical modifications to the fibers, such as silanization or the use of compatibilizers, can be applied, as well as adjustment of the polymer matrix formulation to improve chemical affinity and load transfer between the constituents.

From an industrial processing perspective, the manual rolling used in this research is limited in terms of automation and reproducibility. To overcome this limitation, the development of production routes compatible with industrial techniques such as hot rolling, compression molding, or vacuum infusion would be a necessary step.

Table 3 compares the depth of trauma (BFS—Backface Signature) in different epoxy composites reinforced with natural fibers after ballistic testing, using as a reference the limit of 44 mm established by NIJ 0101.06, above which the level of protection is considered insufficient, even without projectile perforation.

The composites reinforced with raffia fabric (ER10, ER20, and ER30) presented different performances according to the volume fraction of fiber used. The ER10 composite, with 10% raffia, presented an average depth of 38.34 ± 0.29 mm, being below the critical limit and, therefore, demonstrating satisfactory ballistic performance. This behavior can be attributed to a better impregnation of the epoxy matrix in the raffia fabric, allowing a more efficient energy dissipation. However, as the fiber content increases to 20% (ER20) and 30% (ER30), the BFS values increase to 46.82 ± 1.73 mm and 58.40 ± 0.63 mm, respectively, exceeding the limit value and indicating loss of ballistic performance. This behavior may be related to poor fiber distribution, agglomerations, and deficiencies in interfacial adhesion, which compromise the structural integrity and impact absorption capacity of the composites.

On the other hand, the composites reinforced with carnauba fibers (EC20, EC30, and EC40) presented superior performance. All BFS values were below 44 mm, with emphasis on EC40 (31.20 ± 2.90 mm), which presented the smallest depth within this group. The observed trend indicates that, in this case, the increase in fiber concentration did not compromise performance, possibly due to the greater rigidity of the carnauba fibers and their good compatibility with the epoxy matrix, resulting in greater efficiency in dissipating impact energy.

The composites with titica vine fibers (ETVF20 and ETVF40) presented the best results among all the materials analyzed, with average values of 25.57 ± 2.89 mm and 32.51 ± 4.98 mm, respectively. These values demonstrate excellent resistance to ballistic trauma, suggesting that these fibers have high tenacity and a structure favorable to energy dissipation, even with higher reinforcement contents.

In general, the ballistic performance sequence, based on strictly ascending BFS values (lower is better), is: ETVF20 < EC40 < ETVF40 < EC30 < EC20 < ER10 < ER20 < ER30. These results show that the type of fiber used exerts a significant influence on the impact response, and that increasing the volume fraction is not always associated with improved performance. In addition to the aspect ratio, factors such as fiber morphology, interfacial adhesion, and distribution in the matrix must be carefully optimized. Titica vine and carnauba fibers have shown great promise for application in multilayer armor systems, while raffia, despite its good performance at low concentrations, requires strict control of the formulation to ensure its effectiveness.

To make the literature comparison explicit, we quantify the differences in BFS relative to epoxy composites reinforced with carnauba fibers (EC20/EC30/EC40) and titica vine fibers (ETVF20/ETVF40) reported under comparable NIJ conditions. The ER10 laminate (38.34 mm) is higher than EC20/EC30/EC40 (34.90/32.80/31.20 mm) by +9.90%/+16.90%/+22.90%, and higher than ETVF20/ETVF40 (25.57/32.51 mm) by +49.90%/+17.90%, respectively. While ER10 remains NIJ-compliant (≤44 mm), ER20 and ER30 exceed the limit, unlike the cited EC/ETVF systems. These quantitative gaps are consistent with microstructural factors discussed in Section 3.3 (fiber morphology, wet-out, and interfacial adhesion), suggesting that raffia’s performance is competitive only at a lower volume fraction (10 vol%), where controlled pull-out and interfacial sliding occur without excessive voids/delamination. From an application standpoint, raffia laminates are therefore promising as hybrid/sacrificial intermediate layers to balance sustainability and cost with trauma control.

The lower ballistic efficiency of raffia/epoxy compared with carnauba- and titica-reinforced epoxies appears to be multi-factorial. First, bundle morphology and linear density: raffia bundles are typically wider and less uniform, which at higher Vf increases resin-starved contacts and fosters void connectivity, reducing crack-bridging and stress redistribution. Second, fiber–matrix interface: our raffia fabric was used without surface treatment, and SEM reveals interfacial gaps and extensive pull-out, consistent with weaker adhesion than reported for the carnauba/titica systems under comparable epoxy matrices (Table 3). Third, hygroscopicity and surface chemistry (lignocellulosic constituents) may locally plasticize the interface, further lowering shear transfer during impact. Fourth, weave/crimp effects: the raffia fabric’s crimp and yarn packing can reduce in-plane stiffness and magnify through-thickness shear, favoring delamination. Taken together, these factors promote a failure-mode shift, from controlled pull-out/sliding (energy-dissipative) to matrix cleavage + interlaminar delamination + plugging, which explains the larger BFS observed for raffia, especially at 30 vol%.

For reference, multilayered armor systems incorporating aramid laminates (Kevlar™) or ultra-high molecular weight polyethylene (UHMWPE) as the intermediate layer typically present backface signature (BFS) values between 25 and 35 mm under NIJ Level III conditions [18,21,22]. Although the raffia-reinforced epoxy composites developed in this study did not reach BFS values as low as these advanced synthetics, the ER10 configuration (38.34 ± 0.29 mm) successfully remained within the NIJ 0101.06 safety threshold (≤44 mm), confirming its functional ballistic performance. Importantly, raffia-based laminates exhibit an average density of ~1.10 g/cm^3^, lower than that of Kevlar™ (1.44 g/cm^3^) and comparable to UHMWPE (0.97 g/cm^3^), highlighting their potential in lightweight applications. Considering that reduced mass translates directly into improved comfort and mobility for users, the lower density and cost of raffia composites add practical advantages to their performance. Beyond weight considerations, raffia also offers a distinct sustainability advantage: it is a renewable, biodegradable, and locally available natural resource in tropical regions, fostering regional bioeconomy development while reducing dependence on expensive, imported aramid fabrics. From an application standpoint, raffia composites may be employed not as a full replacement of synthetics but as hybrid or sacrificial layers in MAS configurations, acting synergistically with Kevlar™ or UHMWPE to balance impact resistance, weight reduction, cost-effectiveness, and environmental responsibility. This balance between technical feasibility, sustainability, and socioeconomic impact strengthens the case for raffia as a promising candidate for next-generation eco-friendly ballistic systems.

### 3.2. Statistical Analysis

Table 4 presents the results of the analysis of variance (ANOVA) applied to the ballistic test with ballistic clay trauma for the epoxy composites reinforced with raffia fabric: ER10, ER20, and ER30. The variable of interest is the residual ballistic trauma (generally related to the penetration depth in ballistic clay), with the aim of verifying whether there are statistically significant differences between the three groups tested.

The results indicated that the treatments, i.e., the different proportions of raffia fabric, presented 2 degrees of freedom, with a sum of squares of 1014.60 and a mean square of 507.30. The residue presented 12 degrees of freedom, a sum of squares of 14.31, and a mean square of only 1.19. The calculated F value (Fcalc = 425.28) was extremely higher than the tabulated critical value (Ftab = 3.88), indicating a statistically significant difference between the groups with a very high confidence level (*p* < 0.001). This result suggests that the fiber content has a marked effect on ballistic performance, with low variability between the replicates of each group (as demonstrated by the reduced residual mean square). The significant difference can be attributed to the direct influence of the amount of reinforcement on the energy absorption capacity of the composites, highlighting the importance of optimizing the fiber content in the development of materials for ballistic protection.

The application of multiple comparison tests, such as Tukey’s test, was done to identify which pairs of groups differ significantly from each other, as shown in Table 5.

Table 5 presents the results of the Tukey multiple comparisons test applied to the mean values obtained in the ballistics tests with trauma in ballistic clay for the ER10, ER20, and ER30 composites reinforced with raffia fabric. Based on the minimum significant difference (d.m.s.) of 1.84, it was possible to verify that all the differences between the pairs of samples were statistically significant: ER10 versus ER20 presented a difference of 8.48; ER10 versus ER30, a difference of 20.06; and ER20 versus ER30, a difference of 11.58—all values higher than the established threshold. This indicates that there are significant distinctions between the ballistic performances of the three composites evaluated. Based on the observed averages for penetration depth, the ER10 composite presented the lowest ballistic trauma, i.e., the lowest indentation depth after impact, demonstrating the best performance among the groups analyzed. The second-best performance was obtained by the ER20 composite, followed by ER30, which presented the highest trauma. These results suggest that, although increasing the reinforcement content with raffia fabric may favor certain energy dissipation mechanisms, when in excess, as in the case of 30% by volume, it may compromise the structural integrity of the composite due to failures such as poor impregnation, delamination, or increased voids, which impair the ballistic efficiency of the material. Thus, the Tukey test confirms the existence of statistically significant differences between the composites and highlights the importance of seeking an ideal reinforcement proportion, which maximizes energy dissipation without compromising the cohesion of the matrix-fiber system.

### 3.3. Fracture Surface Analysis

**Figure 6:**
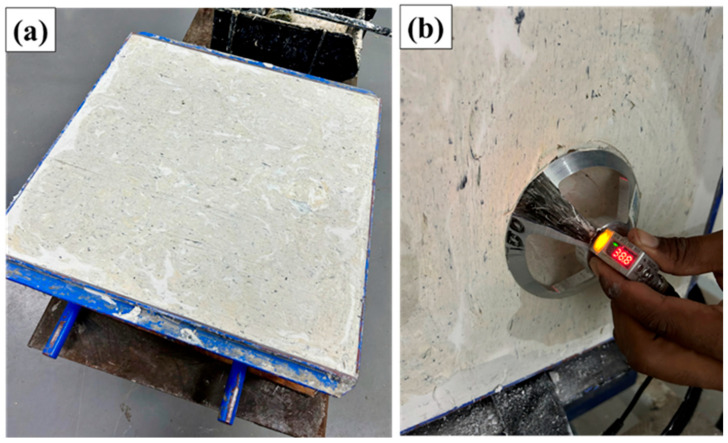
Ballistic response assessment procedures: (**a**) ballistic clay positioned on the impact support, used as a means to record penetration depth and residual trauma; (**b**) measurement of cavity depth in the clay with a laser depth sensor after firing, allowing quantification of the deformation induced by the projectile impact.

Figure 6 illustrates the ballistic impact test performed on the composites.

For ER10, individual BFS values ranged from 38.00 to 38.80 mm (Table 2), with a mean of 38.34 ± 0.29 mm (Table 3), all of which were below the NIJ 0101.06 limit of 44 mm. This result demonstrates that the composite presented sufficient energy absorption capacity to reduce impact transfer to the support material (ballistic clay), remaining within the limit considered safe to avoid lethal risk from internal trauma.

**Figure 7:**
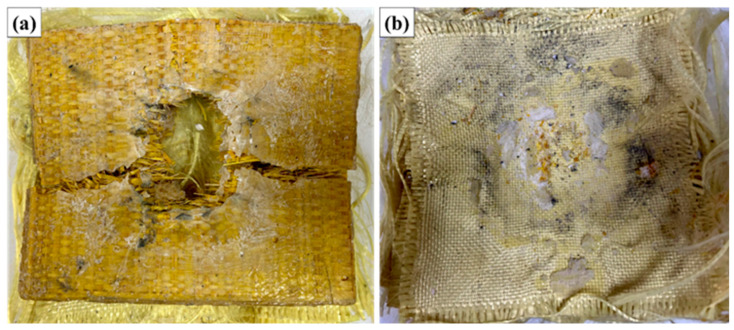
Appearance of the MAS with 10% fiber volume (ER10) after ballistic testing with 7.62 mm ammunition: (**a**) impact face; (**b**) internal face.

Figure 7 illustrates the appearance of the MAS with 10% fiber volume (ER10) after ballistic testing with 7.62 mm ammunition.

Analysis of the images obtained after the ballistic test shows that the ER10 composite panel suffered localized damage in the impact region, with evident fracture of the epoxy matrix and partial rupture of the raffia fabric. Radial cracks and delaminations were observed around the impact zone, typical of energy dissipation mechanisms in laminated composites. Despite the presence of fractures in the matrix and natural fiber network, the structural integrity of the plate was maintained. There was no complete perforation of the armor, and the aramid fabric on the back face remained functional, contributing to the containment of projectile fragments and limiting damage.

Furthermore, the measurement of the indentation depth in the ballistic clay revealed average values lower than 44 mm, meeting the criteria established by the NIJ 0101.06 standard. Therefore, the performance of the ER10 composite can be classified as satisfactory with regard to ballistic protection against 7.62 mm caliber projectiles, within the parameters evaluated. From a macroscopic point of view, the energy absorption mechanisms were predominantly: rupture of the raffia fibers by shear and traction, brittle fracture of the polymer matrix, delamination between the composite layers, and energy absorption in the aramid fabric layer at the back of the armor system. These mechanisms, acting synergistically, allowed the dissipation of the kinetic energy of the projectile, resulting in the preservation of the integrity of the armor and trauma levels within the limits acceptable for user safety.

Figure 8 illustrates the appearance of the MAS with 20% fiber volume (ER20) after ballistic testing with 7.62 mm ammunition.

Visual inspection of sample ER20 after ballistic testing reveals a high level of structural damage, characterized by extensive fracture of the polymer matrix, evident delamination, and rupture of the raffia fibers in the central region of the impact. The formation of a predominantly radial fracture is clearly observed, starting from the point of impact, which propagates in a pronounced manner along the fabric, indicating intense interlaminar failure. The presence of large longitudinal and transverse cracks suggests that the energy dissipation mechanism was strongly associated with crack propagation in the matrix, delamination between layers, and fiber rupture. In addition, it is possible to note a central opening, which corresponds to the site of maximum stress concentration, where there was significant detachment of the epoxy matrix, leaving the fibers partially exposed.

In the rear view of the sample (aramid side), the deformation of the system is still quite visible, but there is no evidence of complete perforation. This indicates that, despite the high level of damage in the intermediate layer (raffia/epoxy), the aramid fabric layer maintained its function of containing the fragments and the projectile, contributing decisively to the overall integrity of the multilayer system. The severity of the damage is directly correlated with the quantitative indentation result recorded on the ballistic clay (average of 46.82 mm), which exceeds the maximum limit of 44 mm established by NIJ 0101.06, classifying the ballistic performance of the ER20 configuration as unsatisfactory for the safety parameters established for trauma. This result suggests that the 20% volumetric fraction of raffia fabric was not sufficient to ensure adequate energy dissipation, with a significant loss of ballistic efficiency when compared to the behavior observed in the ER10 configuration.

Therefore, the macroscopic analysis reinforces the quantitative data obtained in the test, showing that, for this reinforcement proportion, there was an unfavorable combination between rigidity, toughness, and interfacial adhesion, which culminated in a more pronounced structural collapse of the system under ballistic impact.

Figure 9 illustrates the appearance of the MAS with 30% fiber volume (ER30) after ballistic testing with 7.62 mm ammunition.

Visual evaluation of the ER30 samples after ballistic testing revealed extensive localized damage in the impact region, characterized by significant fracture of the epoxy matrix and marked delamination of the raffia fabric layers. Complete rupture of the front layer of the armor is clearly observed, evidenced by the propagation of radial cracks and the removal of matrix fragments in the central region, typical of failure mechanisms associated with brittle fracture of the resin combined with tensional rupture of the fibers.

Furthermore, analysis of the rear face (side in contact with the ballistic clay) shows accumulation of debris and evident textile deformation in the layers of aramid fabric, corroborating that there was a significant transfer of kinetic energy to this region. The presence of abrasion marks, burned regions, and textile collapse zones indicates that the projectile transferred high energy to the structure, resulting in progressive failures.

The failure pattern is characterized by a predominantly plugging type of failure, with expulsion of material in the central region, combined with fiber pull-out mechanisms, interlaminar fracture (delamination), and transverse rupture of the raffia fibers. This condition is particularly noticeable by the exposure of broken and misaligned fibers, in addition to the presence of a well-defined circular central cavity.

However, unlike the ER10 conditions, which maintained structural integrity and presented indentation below the critical limit of the NIJ 0101.06 standard (44 mm), and even the ER20 condition, which presented intermediate behavior, the ER30 condition presented critical failure in both the front and intermediate layers, failing to adequately dissipate the impact energy, as evidenced by the high indentation depth in the ballistic clay (58.4 mm).

This result suggests that an excessive increase in the volumetric content of raffia fabric compromises the ability of the epoxy matrix to effectively transfer and dissipate projectile energy, leading to a more fragile structure that is susceptible to catastrophic fracture. This behavior may be related to the increase in void content, poor impregnation of the matrix, reduction in the overall toughness of the system, and loss of adhesion at the fiber/matrix interface, factors that are recurrent in composites with a high content of fibers of natural origin.

Prior to ballistic testing, scanning electron microscopy (SEM) was carried out on untested raffia/epoxy laminates to establish baseline microstructural features. The ER10 composite exhibited well-impregnated raffia bundles with minimal voids and close contact between the fibrillated raffia surfaces and the epoxy matrix. The rough, microfibrillated morphology of the raffia fibers favored mechanical interlocking and partial wetting by the resin, promoting frictional sliding and energy dissipation during impact. In contrast, ER20 and ER30 laminates displayed localized voids, incomplete wetting, and resin-starved regions between dense raffia bundles—defects that limit interfacial adhesion and load transfer. These as-fabricated microstructural differences are consistent with the observed increase in BFS values and the greater variability for higher fiber fractions. Additional pre-impact morphological details for these materials can be found in Silva et al. [19] and [25], which complement the baseline characterization presented here.

After ballistic impact, SEM observations revealed significant morphological evolution. The impacted ER10 surface showed extensive fiber pull-out, progressive interfacial debonding, and resin tags adhering to fiber surfaces—features indicative of controlled frictional energy dissipation. In ER20 and ER30, the dominant mechanisms shifted toward matrix cleavage, interlaminar delamination, and localized fiber rupture, reflecting insufficient interfacial adhesion and a transition from controlled pull-out to catastrophic separation. Comparison between pre- and post-impact morphologies thus confirms that the fiber–matrix interface plays a decisive role in the ballistic response: a well-wetted, moderately reinforced laminate (ER10) promotes distributed microcracking and efficient frictional energy absorption, whereas excess fiber content leads to void coalescence, delamination, and reduced ballistic efficiency. These results establish a clear structure–property relationship linking microstructural integrity to trauma depth, reinforcing the importance of optimized impregnation and interface quality for high-velocity impact applications.

Figure 10 shows the SEM micrographs of the MAS with 10% raffia fabric.

SEM micrographs of the raffia fabric-reinforced epoxy composite (ER10), obtained at different magnifications (100× and 2400×), reveal a complex microstructure that directly influences its ballistic performance. Fibrillated raffia fiber bundles and rough surfaces are observed, which favor mechanical anchoring and promote additional energy dissipation mechanisms, such as fiber pull-out and progressive interfacial detachment. The epoxy matrix exhibits brittle fracture regions, with smooth surfaces alternating with rough zones associated with shear, characterizing a hybrid failure mechanism.

The SEM features in ER10 (Figure 10) indicate a failure mode that is favorable for energy dissipation: (i) extensive fiber pull-out with visible fibrillation and long sliding paths; (ii) rough matrix fracture with shear lips and hackle marks, consistent with mixed-mode cracking rather than pure cleavage; and (iii) resin tags/imprints on fiber surfaces, evidencing partial adhesion followed by controlled debonding. Together with the limited void content and adequate wet-out at 10 vol%, these features maximize frictional sliding, crack deflection, and crack-bridging, thus reducing BFS. In contrast, the macroscopic response of ER20/ER30 (Figure 8 and Figure 9) is dominated by continuous delamination planes, plugging-type material removal, and powdery matrix cleavage, which are typical of resin-starved and inter-bundle debonding regimes. Such regimes shorten sliding distances, diminish frictional work, and concentrate through-thickness shear mechanisms that explain the higher trauma depths for 20% and 30% compared to 10%.

Although the present work did not experimentally evaluate processing modifications, the SEM observations suggest that some strategies, such as vacuum-assisted impregnation, mild alkaline or silane surface treatments, and post-curing optimization, could be explored in future studies to mitigate porosity and improve fiber–matrix adhesion. These approaches are therefore proposed as prospective methods for further improving interfacial uniformity and structural reliability, rather than as confirmed results of this investigation.

The microstructural examination revealed a complex and heterogeneous morphology in the raffia-reinforced laminates. At lower magnifications, extensive fiber pull-out regions and interfacial gaps were observed, suggesting localized decohesion between the raffia bundles and the epoxy matrix during high-velocity impact. These voids are indicative of interfacial delamination mechanisms, which contribute to energy dissipation by frictional sliding and progressive fiber debonding. Higher magnification images showed broken raffia fibrils with tapered ends and evidence of fibrillation, confirming that tensile and shear rupture as predominant failure modes. The rough fracture surfaces of the epoxy matrix surrounding the fibers suggest a mixed-mode fracture behavior, combining brittle matrix cracking and ductile deformation near the fiber–matrix interface. Additionally, resin-rich areas and partially wetted fiber surfaces denote regions of incomplete impregnation, consistent with the observed decrease in ballistic efficiency for higher fiber fractions (ER20 and ER30). These observations corroborate the macroscopic findings, demonstrating that optimized fiber wetting and controlled interfacial adhesion are essential for maximizing energy absorption and maintaining laminate integrity under ballistic impact.

## 4. Conclusions

The analysis of the results obtained in this study clearly demonstrates that the incorporation of raffia fabric in epoxy matrix composites can be a promising strategy for lightweight ballistic applications, provided that the optimum limits of reinforcement volume fraction are strictly observed. Specifically, the composite containing 10% raffia fabric (ER10) presented satisfactory ballistic performance, meeting the safety criterion established by NIJ 0101.06 (indentation ≤ 44 mm), which highlights its viability as a replacement for conventional synthetic laminates, such as Kevlar™, in multilayer armor (MAS) systems. Interestingly, it was observed that higher fiber fractions (ER20 and ER30) compromised the integrity of the protection, which can be attributed to the increase in microstructural heterogeneity, intensification of interfacial failures, and difficulty in impregnating the matrix, leading to the formation of brittle zones and reduction in the energy dissipation capacity of the composite.

Scanning Electron Microscopy (SEM) analyses were essential to elucidate the failure mechanisms involved, revealing phenomena such as delamination, rupture, and fiber pullout, as well as failures at the fiber-matrix interface. These mechanisms, often associated with poor interfacial adhesion or high reinforcement concentration, explain the inferior mechanical behavior of composites with higher raffia contents. Thus, the observed microstructure reinforces the importance of controlling the fiber proportion and interface quality to ensure the functional performance of the composite under ballistic impact.

This work, therefore, contributes significantly to the advancement of research on sustainable materials for personal protection, demonstrating that natural composites can not only compete with synthetic solutions in terms of performance but also add environmental and economic value. As a future perspective, it is worth highlighting the need to explore chemical surface treatments for raffia fibers, which can improve interfacial adhesion and allow the use of higher reinforcement contents without compromising ballistic resistance. Likewise, the development of numerical models using finite elements may provide important subsidies for structural optimization and performance prediction under different impact conditions. Finally, the evaluation of environmental durability is essential to ensure the applicability of these materials in real-world conditions, thereby guaranteeing their reliability in diverse operational scenarios. Thus, this study establishes a solid basis for the development of ballistic composites of natural origin with high performance and integrated sustainability.

## Figures and Tables

**Table 1:** Nomenclature of the composites utilized in this work.

Nomenclature	Composition
ER10	10 vol.% Raffia fiber fabric
ER20	20 vol.% Raffia fiber fabric
ER30	30 vol.% Raffia fiber fabric

**Table 2:** Individual values, mean, and standard deviation of the backface signature (BFS) obtained for MAS with raffia fabric at volumetric fractions of 10% (ER10), 20% (ER20), and 30% (ER30).

	ER10	ER20	ER30
BFS (mm)	38.40	49.40	57.60
	38.00	45.80	58.80
	38.80	47.81	58.27
	38.26	45.42	58.12
	38.24	45.69	59.22
Mean	**38.34**	**46.82**	**58.40**
Standard Deviation	**0.29**	**1.73**	**0.63**

Note: Means and standard deviations are reported with two decimals; SD is the sample standard deviation (unbiased, *n* − 1), *n* = 5 shots per condition.

**Table 3:** Mean values and standard deviation of the backface signature (BFS) for MAS with raffia fabric (ER10, ER20, ER30) and for different epoxy composites reinforced with natural fibers from the literature.

Samples	BFS (mm)	References
ER10	38.34 ± 0.29	PW *
ER20	46.82 ± 1.73	
ER30	58.40 ± 0.63	
EC20	34.90 ± 2.60	[32]
EC30	32.80 ± 5.70	
EC40	31.20 ± 2.90	
ETVF20	25.57 ± 2.89	[34]
ETVF40	32.51 ± 4.98	
BFS Lethal	44	[33]

PW *—Present Work; EC—Epoxy Carnauba; ETVF—Epoxy Titica Vine Fiber.

**Table 4:** ANOVA of the ballistic test with trauma in ballistic clay of MAS ER10, ER20, and ER30.

Causes of Variation	Degrees of Freedom	Sum of Squares	Mean Square	F_Calc_	F_Tab_
Treatments	2	1014.60	507.30	425.28	3.88
Residue	12	14.31	1.19		
Total	14	1028.91			

**Table 5:** Comparison between the averages (d.m.s) obtained from the average ballistic test values for the evaluated samples.

Samples	ER10	ER20	ER30
**ER10**	0	8.48	20.06
**ER20**	8.48	0	11.58
**ER30**	20.06	11.58	0

## Data Availability

The original contributions presented in the study are included in the article; further inquiries can be directed to the corresponding author.

## DURC Statement

The current research is limited to the academic field of materials science and engineering, specifically the study of sustainable polymeric composites reinforced with natural fibers. Its primary purpose is to advance knowledge on environmentally friendly alternatives for composite materials, contributing to scientific progress, sustainability, and potential civilian protective applications. This work does not pose a threat to public health or national security. The authors acknowledge the dual-use potential of research involving ballistic testing of composite laminates and confirm that all necessary precautions have been taken to prevent potential misuse. As part of our ethical responsibility, we strictly adhere to relevant national and international laws regarding Dual-Use Research of Concern (DURC). We further advocate for responsible deployment, ethical considerations, regulatory compliance, and transparent reporting to mitigate misuse risks and foster beneficial outcomes.
